# Shifting paradigms in primary aldosteronism: reconsideration of screening strategy via integrating pathophysiological insights

**DOI:** 10.3389/fendo.2024.1372683

**Published:** 2025-01-14

**Authors:** Takumi Kitamoto, Yutaro Ruike, Hisashi Koide, Kosuke Inoue, Yoshiro Maezawa, Masao Omura, Kazuki Nakai, Yuya Tsurutani, Jun Saito, Katsuhiko Kuwa, Koutaro Yokote, Tetsuo Nishikawa

**Affiliations:** ^1^ Department of Diabetes, Metabolism and Endocrinology, Chiba University Hospital, Chiba, Japan; ^2^ Department of Social Epidemiology, Graduate School of Medicine, Kyoto University, Kyoto, Japan; ^3^ Endocrinology and Diabetes Center, Yokohama Rosai Hospital, Yokohama, Japan; ^4^ National Metrology Institute of Japan, National Institute of Advanced Industrial Science and Technology, Tsukuba, Japan

**Keywords:** primary aldosteronism, aldosterone measurement, screening test, low renin hypertensive, somatic mutation

## Abstract

Several decades have passed since the description of the first patient with primary aldosteronism (PA). PA was initially classified in two main forms: aldosterone-producing adenoma (APA) and idiopathic hyperaldosteronism (IHA). However, the pathogenesis of PA has now been shown to be far more complex. For this reason, the traditional classification needs to be updated. Given the recent advancements in our understanding of PA pathogenesis, we should reevaluate how frequent PA cases are, beginning with the reconstruction of the screening strategy. Recent studies consistently indicated that PA has been identified in 22% of patients with resistant hypertension and 11% even in normotensives. The frequency is influenced by the screening strategy and should be based on understanding the pathogenesis of PA. Progress has been made to promote our understanding of the pathogenesis of PA by the findings of aldosterone driver mutations, which have been found in normotensives and hypertensives. In addition, much clinical evidence has been accumulated to indicate that there is a spectrum in PA pathogenesis. In this review, we will summarize the recent progress in aldosterone measurement methods based on LC-MS/MS and the current screening strategy. Then, we will discuss the progress of our understanding of PA, focusing on aldosterone driver mutations and the natural history of PA. Finally, we will discuss the optimal strategy to improve screening rate and case detection.

## Search strategy and selection criteria

We searched MEDLINE for articles published from 1 January 1955 to 29 February 2024, using the search terms “primary aldosteronism,” “Conn’s syndrome,’ “hyperaldosteronism,” “screening test,” and “aldosterone measurement”. We mainly focused on English-language publications of the past 5 years (1 June 2019 to 29 February 2024) and selected relevant and highly referenced studies published before this timeframe.

## Introduction

Several decades have passed since the first description of primary aldosteronism (PA) due to aldosterone-producing adenomas (APA) (1). Subsequently, it was observed that some cases of PA lack the classical biological characteristics of hypokalemia or high plasma (or serum) aldosterone concentration (2). Most cases of PA are classified as APA or bilateral adrenal hyperplasia, usually diagnosed as idiopathic hyperaldosteronism (IHA). The former is a surgically curable form of PA, representing more than 5% of patients with hypertension (3), whereas the latter is treated with a mineralocorticoid receptor antagonist (MRA). Additionally, patients with PA exhibit a 1.7- to 3.5-fold increased risk of cardiovascular and cerebrovascular complications (4–7) than essential hypertensives, and early subtype diagnosis is crucial to reversing the excess risk of vascular complications and achieving a better prognosis. To simplify the initial step in diagnosing PA, from 1981, the plasma aldosterone-to-renin activity ratio (ARR) or plasma aldosterone/direct renin ratio (ADRR) has been introduced, given their superiority over the isolated measurements of plasma (or serum) aldosterone concentration and plasma renin activity (PRA) or direct renin concentration (DRC) (8). Since then, ARR and ADRR have played a primary role in screening tests for PA (9–15). In decades, numerous robust prospective studies have established that the prevalence of PA is 3%–19% in all patients with hypertension (3, 16–36), and more patients showed positive results of screening tests in referral centers than in primary care clinics (3, 22, 30, 37). Several models have demonstrated that screening all patients with resistant hypertension for PA is cost-effective (38–40). However, merely less than 2%–5% of patients expected to have PA had been screened (41–44). The factors contributing to this low screening rate may vary across countries. However, the following points seem to be shared: 1) low awareness of PA, 2) the difficulty of changing medications before screening tests, and 3) the need to consider dietary salt intake and the position for blood collection. All these factors contribute to the complexity of the screening phase.

Therefore, this review aimed to restructure the case detection strategy for PA based on numerous novel discoveries over the last few decades, particularly aldosterone driver mutations and the natural history of PA. Moreover, we aimed to discuss an alternative screening strategy that focuses on renin suppression as a biomarker of PA.

## Pathophysiology of PA

PA is a state of hypertension caused by inappropriate aldosterone secretion. Here, “inappropriate” refers to an inappropriate secretory response to salt intake. The body maintains the sodium and fluid balance through the renin–angiotensin–aldosterone system (RAAS) in response to salt intake. PA is a state of hypertension caused by inappropriate aldosterone secretion in response to salt intake, which is independent of renin secretion. Renin secretion is affected by 1) salt restriction (45), 2) fluid volume depletion with diuretics, 3) the use of RAAS inhibitors, such as MRA, angiotensin-II receptor blockers (ARB), and angiotensin-converting enzyme inhibitors, and 4) other hormones, such as glucocorticoids, estrogens, and progestogens (46, 47). Other factors that increase renin levels include renovascular hypertension and pregnancy (high levels of progesterone antagonize aldosterone action in the mineralocorticoid receptor [MR]). Factors suppressing renin secretion include renal failure, β-adrenergic blockers, a-methyldopa, clonidine, and nonsteroidal anti-inflammatory agents. Antidepressants such as selective serotonin reuptake inhibitors elevate aldosterone and renin; however, whether this results in lowering ARR is debatable (48, 49). An overview of the clinically significant factors affecting aldosterone, renin, and ARR levels is summarized in **Table 1**.

**Table 1:** Parameters affecting aldosterone, renin, and ARR.

Parameters	Dietary sodium restriction	Hypokalemia	β-Adrenergic blockers	K^+^-wasting diuretics	K^+^-sparing diuretics	ACE inhibitors	ARBs	Ca^2+^ blockers	α-Blockers	ENaC inhibitors	MR antagonist
Aldosterone	↑	↓	↓	→/↑	↑	↓	↓	→/↓	→	↑	↑
Renin	↑↑	→/↑	↓↓	↑↑	↑↑	↑↑	↑↑	↑	→	↑↑	↑↑
ARR	↓	↓	↑	↓	↓	↓	↓	↓	→	→/↓	→/↓

ARR, aldosterone to renin ratio; ACE, angiotensin-converting enzyme; ARBs, angiotensin II type 1 receptor blockers; ENaC, epithelial sodium channel; MR, mineralocorticoid receptor

[Adapted from *Funder JW*, et al. *J Clin Endocrinol Metab. 2016;101 (*5)*:1889-1916 (*9*).; Naruse M*, et al. *Endocrine Journal. 2022;69 (* 4)*:327-359 (* 15 *)*]

↑, Elevated; ↓, Suppressed; →, Not affected.

In PA, stimulation of renin secretion is blunted because of the feedback effects of aldosterone hypersecretion. In other words, the renin levels remained relatively low in response to these stimuli (50). Regardless of the fluctuations in aldosterone concentration, extracellular fluid volume expansion persists, resulting in continuous renin suppression. If renin-independent aldosterone excess persists, the distal nephron will reabsorb sodium into the body, and potassium will flow out, resulting in hypertension and hypokalemia as the typical PA phenotype.

## Reliable methods for aldosterone measurement

Since screening tests rely on plasma aldosterone and renin levels, measurement reliability is crucial for interpreting clinical outcomes.

The reliability of routine tests depends on the measurement performance. Among the common methods for measuring aldosterone, radioimmunoassays (RIA), liquid chromatography–mass spectrometry (LC-MS/MS), and chemiluminescent enzyme immunoassays (CLEIA) are widely used because of their unique strengths and limitations. RIA, a long-established method, offers practicality and ease of use; however, it suffers from cross-reactivity and variability due to low antibody specificity. LC-MS/MS, which is considered the gold standard, provides unparalleled accuracy and sensitivity (51–53), yet its high cost, time demands, and technical requirements limit its feasibility for high-throughput testing. CLEIA improves upon RIA, with better specificity and compatibility with standardized reference materials, making it suitable for routine clinical use. Each method plays a valuable role in the clinical and research settings, catering to different accuracy and accessibility requirements.

We have undertaken standardization of aldosterone concentration measurements traceable to the International System of Units. We have assembled a Certified Reference Materials (CRM) and a Designated Comparison Method (DCM) (54). A new CLEIA-based test kit, approved for *in vitro* diagnostics, was established using these standards and demonstrated alignment with LC-MS/MS results, supporting CLEIA’s reliability as a routine method (55). Despite variations in RIA due to antibody specificity (55), LC-MS/MS provided a stable reference.

Notably, the median LC-MS/MS value was 48.5 pg/mL compared with 120 pg/mL of RIA (SPAC-S^®^), prompting a proposal to lower the ARR screening cutoff to 55 pmol/mU (PAC_LC-MS/MS_/DRC) compared with 70 pmol/mU (PAC_RIA_/DRC) and set a cutoff value after saline infusion test to 83 pmol/L (PAC_LC-MS/MS_) (56, 57).

Two types of renin measurement, PRA and DRC, are currently available (9). Both PRA and DRC have methodological limitations. First of all, it is important to highlight that low renin activity could not be accurately measured in the specimens obtained from patients with PA (58).

In particular, PRA is dependent on the generation of angiotensin I, which can result in significant variability in low-renin states owing to reduced renin secretion (58). This often leads to an underestimation of renin activity (59). However, DRC provides a more stable measurement, as it directly quantifies renin concentration without relying on angiotensin I production (60). Consequently, the DRC is less affected by fluctuations in substrate levels and remains relatively stable even in low-renin states. In contrast, the PRA tends to show greater variability and is more susceptible to pre-analytical errors (61).

The poor correlation between the PRA and DRC in low-renin states is particularly relevant for PA screening (62). In such cases, PRA may underestimate renin levels due to its dependence on angiotensin I, which can affect the ARR used in screening. DRC’s stability makes it a potentially more reliable indicator of renin levels in these cases, thus potentially improving the accuracy of PA screening by reducing false-negative results when ARR is used.

Furthermore, the correlation between the PRA and DRC can be poor, particularly in low-renin states (62). This is because PRA reflects the overall activity of the renin–angiotensin system (RAS) and is more sensitive to feedback control, whereas DRC measures renin levels more directly. This distinction is crucial in PA screening as each method has different implications for accuracy and reliability depending on the renin levels.

Additionally, the variability in the renin substrate among the samples can lead to decreased PRA when the substrate concentration is insufficient for a 90-min reaction (63). If specimens are left at room temperature after collection, angiotensin levels can increase, causing inaccuracies in the PRA. PRA measurements are most accurate when specimens are chilled; however, this can lead to inaccurate DRC values. Therefore, proper handling conditions are essential to obtain reliable renin measurements and improve the overall accuracy of PA screening.

Understanding these possible errors is necessary before testing to interpret the results accurately. PRA measurements are affected by several conditions, resulting in poor reproducibility between laboratories (64–66). The DRC procedure is inexpensive, requires a short test time, and has superior specimen handling and reproducibility. However, PRA is currently the mainstream test because of low correlation of DRC with PRA ≤ 1.0 ng/mL/h. Some studies have demonstrated that DRC showed lower sensitivity than PRA when used as a screening test in a ratio to aldosterone levels compared with PRA (58, 67, 68). Nevertheless, a method that adequately correlates with PRA over a wide range has been developed (69) and may be widely used in the future because of its simplicity.

## The current screening strategy, along with the guidelines

Each guideline from a different country has specific criteria for screening tests. The Japan Endocrine Society (JES) revised its guidelines for PA in 2021 (15). In **Table 2**, we summarize the current statements for screening strategies compared with the Endocrine Society guidelines (9), which have been adopted in many research reports. The Endocrine Society guideline recommends targeted screening for PA in specific high-risk groups, such as patients with resistant hypertension, hypokalemia, or adrenal incidentalomas, as well as those with a family history of early-onset hypertension. This selective approach aimed to efficiently identify PA in patients most likely to have the condition based on clinical indicators. In contrast, the JES guidelines advocate a broader screening approach and recommend testing for all patients with hypertension. This inclusive strategy reflects the findings that PA often presents without hypokalemia (70) and poses a higher cardiovascular risk (7, 71, 72) and that early diagnosis and treatment can be more cost-effective over time (38, 39). By screening all patients with hypertensions, the JES aims to improve PA detection rates and address the underdiagnosis of PA in the general hypertensive population.

**Table 2:** Comparisons of screening strategy between ES and JES guidelines.

	US	Japan
Screening target population	Patients with 1) Sustained BP above 150/100 mm Hg on each of three measurements obtained on different days 2) With hypertension resistant to three conventional antihypertensive drugs (including a diuretic), or controlled BP (<140/90 mm Hg) on four or more antihypertensive drugs 3) Hypertension and spontaneous or diuretic-induced hypokalemia 4) Hypertension and adrenal incidentaloma 5) Hypertension and sleep apnea 6) Hypertension and a family history of early onset hypertension or cerebrovascular accident at a young age (<40 years) 7) All hypertensive first-degree relatives of patients with PA.	A) All hypertensive patients, especially those with a high prevalence of PA B) Clinical features suspicious for PA include 1) Spontaneous hypokalemia 2) Resistant hypertension 3) Hypertension onset before 40 years of age 4) Adrenal tumor 5) Stroke at a young age 6) Sleep apnea syndrome
Preparation	Diet: liberalize sodium intake Potassium replacement: to achieve plasma [K+] of 4.0 mmol/L Antihypertensive agents: Withdraw at least 4 weeks: Spironolactone, eplerenone, amiloride, and triamterene Potassium-wasting diuretics Products derived from licorice root withdraw at least 2 weeks: β-Adrenergic blockers, central α-2 agonists, and non-steroidal anti-inflammatory drugs Angiotensin-converting enzyme inhibitors, angiotensin receptor blockers, renin inhibitors, and dihydropyridine calcium channel antagonists Establish oral contraceptives and hormone replacement therapy when direct renin concentration is measured	Diet: not mentioned Potassium replacement: preferred to normalize potassium level (target value not stated) Antihypertensive agents: Withdraw at least 4 weeks Mineralocorticoid receptor antagonists (spironolactone, eplerenone, esaxerenone) Switching anti-hypertensive medicines to calcium channel blockers, alpha-blockers, or combinations whenever possible
Blood collection	Collect blood 1) After the patient has been up (sitting, standing, or walking) for at least 2 h and seated for 5 min–15 min 2) Carefully, avoiding stasis and hemolysis 3) Maintain sample at room temperature during delivery to laboratory and prior to centrifugation	Collect blood 1) Obtained at any time in the sitting position is acceptable for screening 2) Desirable to conduct blood sampling early in the morning in the supine position after overnight fasting 3)* PRA (in a container of ice), DRC (at room temperature): PRA or DRC less than 30 min of transport to laboratory.

The target population, preparation, and blood collection procedure for PA screening tests recommended in clinical practice guidelines from Endocrine Society (9) and Japan Endocrine Society (15) are summarized. * We added point#3, as handling the samples properly for accurate results is important.

Both guidelines converge on prioritizing high-prevalence groups but differ in the scope of initial screening due to varied emphasis on risk factors, cost-effectiveness, and the broader impact of PA on cardiovascular health.

Each guideline has recommended the use of ARR or ADRR as screening tests (9, 13–15, 73). Since ARR and ADRR are strongly influenced by renin value, the combined value of PAC_RIA_ (≥120 pg/mL in JES, ≥150 pg/mL in the United State [U.S.]) and ARR (≥200 pg/mL per ng/mL/h) or ADRR (≥24 pg/mL per mU/L) are recommended. However, we should be aware that more than 35% of patients with PA, particularly those with bilateral PA, have a PAC_RIA_ < 150 pg/mL (74). PAC_CLEIA_ cutoff is also mentioned in the JES guidelines. They recommended judging the screening test positive when PAC_CLEIA_ ≥ 60 pg/mL and ARR ≥ 200 as positive. An ARR between 100 and 200 is provisionally positive and set as a borderline range until the PAC_CLEIA_ is generalized and its optimal cutoff is established. When the active renin concentration (ARC) is measured instead of PRA, they recommended judging the screening test positive when ARR (PAC_CLEIA_/ARC) ≥40 and PAC ≥60 pg/mL and ARR between 20 and 40 set as a borderline range.

Japanese and U.S. PA experts now specify that screening with normal blood collection is acceptable to enhance the screening rate. Many drugs and conditions do not hinder the detection of typical PA (75). In cases where the initial test is inconclusive or strongly suspicious, adjustment of interfering anti-hypertensive drugs to the ARR/ADRR thresholds (see **Table 2** for the withdrawal period of each drug) and blood collection early in the morning, in the supine position after overnight fasting, are recommended (76–79). Confirmatory tests should be performed to confirm inappropriate aldosterone secretion and to exclude false-negative results. Evidence regarding the number of tests to be performed or the superiority of any confirmatory test is unavailable. When patients desire surgical treatment, performing adrenal venous sampling (AVS) is recommended for the subtype diagnosis. From these comparisons, a general agreement on the issues and methods of screening tests is observed.

## Variable prevalence rates of PA as determined by the ARR screening strategy

Several studies have evaluated the prevalence of PA using the ARR (or ADRR) as a screening strategy, as summarized in **Table 3**. The prevalence of positive screening results was variable across the studies (3, 16–36, 80–82), ranging from 3% to 19% when considering the general hypertensive population and from 21% to 68% in patients with resistant hypertension, whereas the prevalence of confirmed PA cases was 3%–19% and 7%–22%, respectively. The factors causing the variable prevalence of PA are unstandardized screening strategies (i.e., different thresholds, and collection in different positions) and methodological issues (as discussed early). In addition to medications, several factors influence the ARR, potentially impacting on the accuracy of PA screening, such as pulsatility of aldosterone secretion, sodium intake, ethnicity, and posture (**Table 1**). Aldosterone secretion is not constant but rather pulsatile, with natural fluctuations throughout the day and night. One study demonstrated the high reproducibility of the ARR in multiple measurements taken on the same patient over time (83); however, many studies have indicated a large variability in plasma aldosterone levels in patients with or without PA (84–88). Dietary sodium restriction led to a misinterpretation of screening test, with normal results in 52% of patients with PA (89). Ethnic differences can influence the baseline aldosterone and renin levels. Certain ethnic groups, such as African Americans, tend to have lower renin levels (90). Body position affects aldosterone and renin levels. For example, standing increases renin secretion due to decreased renal perfusion, which raises renin levels and lowers the ARR. Prevalence studies have shown a wide range in the proportion of APAs in patients with PA, which impacts the aldosterone and renin values and serum potassium levels in each cohort. These factors can affect ARR measurements and screening accuracy for PA. A recent study using LC-MS/MS or CLEIA showed that more than half of the patients had an ARR ≤ 30 ng/mL/h at least once, below the screening threshold (91). PAC is highly variable, and the boundary between normal and abnormal cannot be determined independently.

**Table 3:** Prevalence of confirmed PA cases and positive screening results in patients with hypertension.

Author (Year, region)	Design	Center	Population	Cases screened (n)	Scr. test	Test position	Conf. test	Subtyping	Positive Scr. test	Positive Conf. test	APA (%)	Aldosterone (ng/dL)	Renin (ng/mL/h)
Lim PO, et al. (2000, UK) (16)	Ret-Sin	Ref	HT	465	PAC/PRA ≥ 27	Seated	FST	n.a.	17%	9%	n.a.	13.3 (10.5–17.7)	0.3 (0.2–0.4)
Loh KC, et al. (2000, Singapore) (17)	Pro-Mul	Pri	HT	350	PAC/PRA > 20, PAC > 15	Seated	SIT	CT/AVS	18.0%	4.6%	50%	22.0 ± 1.1*	0.02–1.04
Calhoun et al. (2002, U.S.) (80)	Pro-Sin	Ref	R-HT	88	U-Ald > 12, PRA < 1.0	Not specified	U-Ald PRA	n.a.	20.0%	20.0%	n.a.	19.2 (5.0–47.0)	0.3 (0.2–0.8)
Rossi E, et al. (2002, Italy) (3)	Pro-Sin	Ref	HT	1,046	CCT, PAC/PRA ≥ 35	Seated	SIT	n.a.	12.8%	6.3%	n.a.	n.a	n.a
Mosso L, et al. (2003, Chile) (19)	Ret-Mul	Pri	HT	609	SAC/PRA > 25	Seated	FST	n.a.	10.2%	6.1%	n.a.	16.9 ± 6.8	0.3 ± 0.2
Stowasser M, et al. (2003, Australia) (20)	Ret-Sin	Ref	HT	300	PAC/PRA > 30	Seated	FST	CT/AVS	20%	18%	31%	21.1 ± 1.6	0.4 ± 0.04
Strauch B, et al. (2003, Germany) (21)	Pro-Sin	Ref	HT	402	PAC/PRA ≥ 50	Upright	SIT	CT/AVS	22%	19%	36%	42.2 ± 36.9	0.2 ± 0.2
Omura M, et al. (2004, Japan) (22)	Pro-Sin	Ref	HT	1,020	PAC > 12, PRA < 1.0	Supine	FUT	CT/AVS	12%	6%	74%	n.a.	n.a.
Nishizaka MK, et al. (2005, U.S.) (23)	Pro-Sin	Ref	R-HT	265	PRA < 1.0	Not specified	PRA, U-Ald	n.a.	58%	22%	n.a.	n.a.	n.a.
Rossi GP, et al. (2006, Italy) (14)	Pro-Mul	Ref	HT	1,125	CCT ¶	Seated	SIT	CT/AVS	20%	11%	63%	29.7 (17.0–226)	0.62 (0.02–0.96)
Fogari R, et al. (2007, Italy) (24)	Pro-Sin	Ref	HT	3,000	PAC/PRA ≥ 25	Upright	SIT	CT	23%	6%	30%	13.6 ± 6.2	0.3 ± 0.2
Douma S, et al. (2008, Greece) (25)	Ret-Sin	Ref	R-HT	1,616	SAC/PRA ≥ 37, SAC ≥ 15	Supine	PRA, PAC	CT/AVS	21%	11%	n.a.	22.9 (15.1–1503)	0.14 (0.01–0.65)
Westerdahl C, et al. (2011, Sweden) (26)	Pro-Mul	Pri	HT	200	SAC/PRC > 2.34	Seated	FST	CT/AVS	18%	6%	27%	11.8 ± 8.7	4.0 ± 2.2†
Sigurjonsdottir HA, et al. (2012, Sweden) (27)	Pro-Mul	Ref, Pri	HT	353	SAC/PRC > 4.61, SAC > 15.5	Seated	OSLT	CT/AVS	13%	6%	60%	24.0 (20.0–33.4)	n.a. †
Sang X, et al. (2013, China) (28)	Pro-Mul	Ref, Pri	R-HT	1,656	PAC/PRA ≥ 20	Seated	SIT	CT/AVS	30%	7%	51%	32.0 (24.0–49.7)	0.4 (0.1–0.6)
Galati SJ, et al. (2016, U.S.) (29)	Pro-Sin	Ref	HT	296	PAC/PRA ≥ 20.0, PAC ≥ 10.0 PRA < 1.0	Seated	OSLT	CT/AVS	5%	1%	n.a.	41, 17‡	0.2, 0.3‡
Monticone S, et al. (2017, Italy) (30)	Pro-Mul	Pri	HT	1,672	SAC/PRA ≥ 30.0, SAC ≥ 10.0	Seated	SIT, CCT	n.a.	14%	6%	27%	31.0 (22.0–42.9)	0.3 (0.2–0.5)
Kayser SC, et al. (2018, Netherlands) (31)	Ret-Mul	Pri	HT	361	PAC/PRC ≥ 4.0, PAC ≥ 40.0	Not specified	SIT	n.a.	26%	3%	n.a.	66.8 ± 11.8	0.5 (0.3–0.7)§
Brown JM, et al. (2020, U.S.) (32)	Ret-Mul	Ref	Nor, HT, R-HT	1,015	PRA < 1.0, < 0.6 (seated/supine),	Seated or supine	OSLT	n.a.	68%	11.3%, 22.0% ||	n.a.	8.3 (6.9–15.0), 25.0 (14.2–38.8) **||**	0.5 (0.2–0.6), 1.1 (0.6–3.1) ||
Burrello, et al. (2020, Italy) (81)	Ret-Sin	Ref	HT	5,100	PAC/PRA ≥ 30.0, PAC ≥ 10.0	Not specified	SIT, CCT	CT/AVS	37%	8%	n.a.	n.a.	n.a.
Parasiliti-Caprino, et al. (2020, Italy) (82)	Ret-Sin	Ref	R-HT	170	Resistant hypertensives	n.a.	SIT	n.a.	40%	19	n.a.	35.8 (24.7–44.2)	0.3 (0.2–0.8)
Xu Z, et al. (2020, China) (33)	Pro-Mul	Pri	HT	1,020	PAC/PRC > 20.0, PAC > 20	Upright	CCT, SIT	CT/AVS	9%	4%	20%	16.5 (13.2–21.5)	3.6 (1.1–6.6) †
Xu F, et al. (2021, China) (34)	Pro-Sin	Ref	HT	7,594	PAC/PRC ≥ 3.7, PAC ≥ 10.0	Upright	SIT, CCT	CT/AVS	5%	3%	39%	n.a.	n.a.
Asbach E, et al. (2022, Germany) (35)	Pro-Mul	Pri	HT	200	SAC/PRC ≥ 12.0, PAC ≥ 5.0	Seated	SIT, CCT	CT/AVS	21%	6%	9%	11.2 (8.3–15.9)	4.4 (2.0–6.5)†
Yoon M, et al. (2022, Korean) (36)	Ret-Sin	Ref	HT	1,173	PAC/PRA ≥ 30 or PAC/PRA > 20, PAC > 15	Not specified	SIT	CT/AVS	31%	6%	27%	25.4 (20.0–32.6)	0.4 (0.3–0.7)

The prevalence of PA in hypertensive patients was summarized.

Scr. test, screening test; Conf. test, confirmatory test; Ret, retrospective analysis; Pro, prospective cohort; Sin, single center; Mul, multicenters; Pri, primary care; Ref, referral center; HT, hypertensive patients; R-HT, resistant hypertensive patients; Nor, normotensive patients; PAC, plasma aldosterone concentration (ng/dL); SAC, serum aldosterone concentration (ng/dL); PRA, plasma renin activity (ng/mL/h); PRC, plasma renin concentration (mIU/L, pmol/L, specified at †, §); FST, fludrocortisone suppression test; SIT, saline infusion test; FUT, furosemide upright test; OSLT, oral salt loading test; CCT, captopril challenge test; U-Ald, urinary aldosterone in a day (µg/24 h) *; Mean ± SE, †; (mIU/L), ‡; Only two cases were diagnosed PA, §; (pmol/L), ||; (normotension), (resistant hypertension), ¶; In the Captopril Challenge Test (CCT), an ARR ≥ 40.0 at baseline, ≥ 30.0 after captopril administration, or a logistic discriminant function (LDF) score ≥ 0.50 indicated a positive result (see details in the original paper). A case was considered to have screened positive for PA if any of these three criteria were met. A SIT was conducted after a positive screening to confirm autonomous aldosterone secretion.

A better screening strategy is required for accurate PA case detection. Numerous discussions are available regarding the need for standardization. However, international agreements on screening tests are yet to be reached. The reasons seem to be the following: 1) matching the measurement system of each institution is almost impossible, 2) setting body position and time for each measurement in patients is challenging in daily practice; and 3) serum or plasma aldosterone cutoff values vary according to measurement conditions and probably according to ethnicity. For example, the *KCNJ5* somatic mutations, an aldosterone driver mutation causing severe forms of PA is known to show large ethnic differences in frequency (92–98). As we will discuss in the next section, we will delve into aldosterone driver mutations, which will advance our understanding of PA’s pathophysiology of PA to better discuss screening strategies.

## Distinctive clinical presentation of PA by aldosterone driver mutations

Our understanding of the pathophysiology of PA has significantly advanced since the discovery of aldosterone driver mutations, which have been observed even in the adrenals of normotensive individuals and APAs and have shown sex and ethnic differences. Somatic mutations in the gene encoding *KCNJ5* in APA (99) cells were first reported in 2011. Following this discovery, *ATP2B3* and *ATP1A1* (100), and *CACNA1D* (101) somatic mutations were identified. Recent work has demonstrated that more APAs carry *CACNA1D* mutations in *KCNJ5* wild-type APAs when CYP11B2 immunohistochemistry-guided high-throughput sequencing is used instead of Sanger sequencing (102). More than 90% of APAs harbored any of these aldosterone driver mutations. Since 2011, several studies have reported the frequency of *KCNJ5* mutations (92–96, 99, 103–119) (**Table 4**). The frequency of *KCNJ5* mutation in APA is higher in eastern countries [70.5 (43.2–74.7) (%) (95, 96, 103–110)] than in western nations [41.0 (35.5–51.8%) (92–94, 99, 102, 111–119)]; however, *KCNJ5* mutation is commonly the dominant mutation across the countries. We previously discussed the clinical impact of *KCNJ5* mutations on APAs (120).

**Table 4:** Prevalence of *KCNJ5* mutation in APAs from Asia, the U.S., and European countries.

Author (Year, region)	Design	APA (n)	*KCNJ5* Seq	*KCNJ5* (%)	*KCNJ5*-mutated APA			*KCNJ5*-wild APA		
					Aldosterone (ng/dL)	Renin (ng/mL/h)	Potassium (mEq/L)	Aldosterone (ng/dL)	Renin (ng/mL/h)	Potassium (mEq/L)
Choi M, et al (2011, Sweden) (99)	Ret-Sin	22	Conv	36.4%	n.a.	n.a.	3.5 ± 0.6	n.a.	n.a.	3.5 ± 0.4
Akerstrom T, et al (2012, Sweden, Germany, Australia, France) (111)	Ret-Mul	348	Conv	45.1%	n.a.	n.a.	n.a.	n.a.	n.a.	n.a.
Azizan EA, et al (2012, UK, Australia) (92)	Ret-Mul	73	Conv	41.1%	n.a.	n.a.	n.a.	n.a.	n.a.	n.a.
Boulkroun S, et al (2012, France, Germany, Italy) (93)	Ret-Mul	380	Conv	33.9%	38.7 (24.9–61.9)	0.30 (0.12–0.49)	4.7 ± 0.6	31.8 (20.1–47.9)	0.27 (0.15–0.60)	5.2 ± 0.4
Taguchi R, et al (2012, Japan) (108)	Ret-Sin	23	Conv	65.2%	6.05 (4.68–9.78)	0.40 (0.30–0.80)	4.2 ± 1.1	4.75 (4.25–7.18)	0.70 (0.45–0.98)	4.5 ± 0.4
Arnesen T, et al (2013, Norway) (113)	Ret-Sin	28	Conv	35.7%	31.2 (21.6–38.3)	0.50 (0.22–0.77)	3.2 (3.0–3.6)	32.3 (24.2–45.2)	0.20 (0.20–0.50)	3.3 (3.0–3.4)
Fernandes-Rosa FL, et al (2014, France, Germany, Italy) (114)	Ret-Sin	474	Conv	38.0%†‡§	29.8 (22.6–41.4)	1.7 (1.0–2.7)*	3.3 (3.0–3.6)	29.3 (18.4–42.4)	1.7 (1.0–3.1)*	3.0 (2.7–3.3)
Kitamoto T, et al (2014, Japan) (105)	Ret-Sin	108	Conv	69.4%†‡§	43.6 (30.0–61.1)	0.2 (0.1–0.4)	3.2 ± 0.5	24.7 (18.5–38.1)	0.2 (0.1–0.5)	3.3 ± 0.5
Williams TA, et al (2014, Italy) (94)	Ret-Mul	112	Conv	39.3%‡§	48.0 (32.0–66.0)	0.2 (0.1–0.3)	2.9 ± 0.7	47.0 (35.0–60.0)	0.2 (0.20–0.39)	3.1 ± 0.7
Akerstrom T, et al (2015, Sweden, Germany, Australia) (112)	Ret-Mul	165	Conv	54.5%	136.5 ± 21.9	n.a.	n.a.	135.5 ± 14.5	n.a.	n.a.
Cheng CJ, et al (2015, Taiwan) (103)	Ret-Sin	69	Conv	37.7%	49.4 ± 29.2	0.45 ± 0.32	2.6 ± 0.6	42.1 ± 29.5	0.66 ± 1.19	2.9 ± 0.6
Scholl UI, et al (2015, U.S., Germany) (119)	Ret-Mul	90	Conv	37.1%†‡§||	n.a.	n.a.	3.1 ± 0.6	n.a.	n.a.	3.4 ± 0.6
Wang B, et al (2015, China) (109)	Ret-Mul	114	Conv	75.4%	25.1 ± 6.8	n.a.	2.9 (2.6–3.2)	18.6 ± 5.5	n.a.	3.5 (3.1–3.9)
Wu VC, et al (2015, Taiwan) (95)	Ret-Mul	148	Conv	59.5%†‡§	59.7 ± 32.9	n.a.	3.2 ± 0.7	40.7 ± 25.1	n.a.	3.8 ± 0.6
Zheng FF, et al (2015, China) (96)	Ret-Mul	168	Conv	76.8%†‡§	36.5 (22.3–47.7)	0.3 (0.1–0.7)	2.6 (2.2–2.8)	31.5 (21.2–44.5)	0.6 (0.2–2.4)	2.9 (2.5–3.0)
Hong AR, et al (2016, Korean) (104)	Ret-Sin	66	Conv	71.2%†‡§	41.3 (31.7–52.5)	0.10 (0.10–0.19)	2.8 (2.5–3.1)	48.2 (33.0–55.2)	0.10 (0.10–0.10)	2.9 (2.6–3.0)
Nanba K, et al (2018, U.S.) (117)	Ret-Sin	75	Cyp11b2-g	42.7%†‡§	n.a.	n.a.	n.a.	n.a.	n.a.	n.a.
Warachit W, et al (2018, Thailand) (110)	Ret-Sin	96	Conv	69.8%	54.9 (33.2–76.5)	0.37 (0.20–0.67)	2.6 ± 0.6	34.7 (24.5–62.9)	0.39 (0.19–0.62)	2.4 ± 0.6
Mohideen SK, et al (2019, Malaysia) (106)	Ret-Sin	54	Conv	31.5%	111.3 ± 169.5	n.a.	2.7 ± 0.8	60.9 ± 41.7	n.a.	2.9 ± 0.7
Nanba K, et al (2019, U.S.) (118)	Ret-Sin	69	Cyp11b2-g	34.2%†‡§	n.a.	n.a.	n.a.	n.a.	n.a.	n.a.
De Sousa K, et al (2020, France) (102)	Ret-Sin	48	Cyp11b2-g	43.8%†‡§||	116.2 (92.6–142.0)	1.0 (1.0–3.2)*	3.1 (2.6–3.2)	93.3 (53.2–150.8)	1.0 (1.0–3.2)*	2.7 (2.1–3.1)
Guo Z, et al (2020, Australia) (115)	Ret-Sin	40	Cyp11b2-g	35.0%†‡§||	99.1 (49.4–167.5)	2.2 (0.9–2.8)*	2.8 (2.6–3.0)	n.a.	n.a.	n.a.
Nanba K, et al (2020, Japan) (107)	Ret-Sin	115	Cyp11b2-g	72.6%†‡§	44.4 (29.6–61.7)	0.2 (0.1–0.4)	n.a.	33.2 (21.8–41.9)	0.2 (0.1–0.3)	n.a.
Meyer LS, et al (2021, Germany) (116)	Pro-Sin	41	Cyp11b2-g	56.1%	n.a.	n.a.	n.a.	n.a.	n.a.	n.a.

The prevalence of *KCNJ5* mutation in aldosterone-producing adenoma (APA) is summarized. Akerstrom T et al. included *CACNA1D* (n = 5), *ATP1A1* (n = 10), and *ATP2B3* (n = 5) mutated APAs in *KCNJ5* wild APAs. Guo Z et al. excluded *CACNA1H* and *CLCN2* mutated APAs from *KCNJ5*-mutated APAs.

Abbreviations are described in the same way as in **Table 2**. The others are following. APA; aldosterone-producing adenoma, Conv; conventional approach, Cyp11b2-g; CYP11B2 guided sequencing, * Plasma renin concentration (mIU/L); *CACNA1D* (†), *ATP1A1* (‡), *ATP2B3* (§), *CTNNB1* (||) mutated APA excluded from *KCNJ5* wild-type APAs

In summary, the typical clinical characteristics of APAs harboring *KCNJ5* mutations are female dominance, higher aldosterone production capacity, and induction of hypokalemia, compared with *KCNJ5*-wild APAs. While the frequency of *KCNJ5* mutations in APAs differs between Asians and Westerners, the plasma aldosterone levels of *KCNJ5*-mutated APAs are similar between the two groups [*KCNJ5* mutated vs. wild APA: PAC_RIA_ 46.9 (40.1–59.8) and PRA 0.3 (0.2–0.4) in Asia, and PAC_RIA_ 48.0 (31.2–116.2) and PRA 0.3 (0.2–0.5) in Western countries] (**Table 4**). The distribution of somatic mutations in APAs might have caused variability in the aldosterone values of patients with PA in these studies. The underlying cause of the distinctive frequency of *KCNJ5* mutations has not yet been elucidated. Whether this is due to a selection bias among patients with APA enrolled in the studies or environmental factors, such as ethnicity, remains to be investigated. In parallel with the research on somatic mutations in APA, efforts have been extended to normotensive and IHA cases. Approximately half of the adrenals from normotensive participants contained aldosterone-producing micronodules (APMs; formerly known as aldosterone-producing cell clusters), termed by CYP11B2-positive clusters (121), and more than 40% of APM harbored *CACNA1D* or *ATP1A1* somatic mutations (122, 123). The most frequent mutation identified in these patients was that of the *CACNA1D* gene, which was found to almost exclusively cause IHA (124), even if this interpretation is limited by the scarcity of surgically resected IHA samples. The differential distribution of somatic mutations, such as *KCNJ5* mutations that appeared uniquely in APAs and *CACNA1D*, which was exclusively observed in IHA, might partly explain the distinctive clinical characteristics of APA and IHA (125). Moreover, APMs in the adrenal glands of normotensive increase with aging (123, 126, 127). These findings support the concept of a continuum pathophysiology of PA from normotensive to participants with hypertension (128). A recent study using expression quantitative trait loci analysis identified the risk loci for PA (129). These discoveries have led us to conceive novel ideas for the methods for early diagnosis of PA (130–132). Aldosterone driver mutations that increase with age have been observed in normotensive patients, and some mutations display sex differences. Furthermore, different mutations demonstrate different clinical behaviors in aldosterone overproduction. In light of these points, defining a certain threshold for absolute aldosterone values for the boundary between PA and non-PA cases should be complicated.

## Natural history of primary aldosteronism

The identification of aldosterone driver mutations in the adrenal glands of normotensive participants also raises the question of whether aldosterone secretion abnormalities occur before the onset of hypertension. The answer to this question will clarify the natural history of PA, allowing us to reconsider when and how screening tests should be performed. A study in 2017 examined 210 normotensive participants with a PRA below 1.0 ng/mL/h, of which 14% were subsequently diagnosed with PA (128). Although no significant difference was observed in the ARR between confirmed PA cases and controls, aldosterone levels were significantly higher in the PA group. Furthermore, even among suspected and unconfirmed PA cases, 20% of them received a confirmed PA diagnosis over 5 years, with one-third of cases showing a unilateral subtype. These results indicated that the pathogenesis of PA is continuous and progressive.

Another finding from these studies is that the ARR may not always accurately reflect the pathogenesis of PA. A recent meta-analysis evaluating the sensitivity and specificity of ARR to detect patients with PA demonstrated a wide variation in sensitivity from 10% to 100% and specificity from 70% to 100% (133). Of note, 3 of 10 studies reported ARR sensitivity of less than 50%, suggesting a limited ability of ARR to adequately identify patients with PA. A recent study used the amount of aldosterone excreted daily in the urine instead of the ARR to detect PA. Using 24-h urinary aldosterone excretion can address diurnal aldosterone variations in a screening test. As salt intake is a major factor in diagnosing PA (89), this study confirmed the salt intake and analyzed cases of renin suppression (32). The results showed that 22% of patients with resistant hypertension and 11% of normotensives had PA. The sensitivity of ARR in this study was less than 30%. Furthermore, a continuum of aldosterone levels and biomarkers of MR activity, such as urinary sodium–potassium ratio, was observed from normotension to hypertension resistance. This finding has been confirmed in a recent elegant study (134). This human physiological study demonstrates a continuum of dysregulated aldosterone production in the low-renin phenotype. Based on a series of studies, we speculated that in patients with PA, dysregulated aldosterone secretion in response to salt leads to renin suppression and demonstrates a continuous and progressive pathophysiology. In the natural history of PA, blood pressure is determined by individual sensitivity, and hypertension occurs when an aldosterone hypersecretion reaches a certain threshold. Suppressed renin seems to be an early biomarker for the detecting PA.

## Emerging evidence on the association of low renin with cardiovascular complications

Whether high aldosterone levels per se cause cardiovascular diseases should be investigated. Extraordinarily high aldosterone levels due to chronic sodium deficiency never induce high blood pressure but rather low or normal blood pressure or any cardiovascular or renal damage (135). Thus, inappropriate aldosterone secretion—inappropriate for salt intake (136)—should be a key player in excessive vascular risk. Renin, receiving a feedback inhibition by aldosterone, may serve as a valuable biomarker for identifying dysregulated aldosterone secretion (137, 138).

A well-designed study has added new evidence regarding the association between suppressed renin, high aldosterone levels, and cardiovascular disease (139). The authors demonstrated an association between serum aldosterone concentration and coronary artery calcium (CAC) scores, a marker of subclinical atherosclerosis, in a multiethnic population without antihypertensive medication. The striking result of their study was that a marked association between aldosterone levels and CAC score and an increased risk of all-cause mortality were observed only among individuals with low renin levels. They also showed that the association between elevated aldosterone levels and subclinical atherosclerosis was only partially mediated by blood pressure, indicating the direct cardiovascular damage of aldosterone independently of hypertension. More recently, one study clarified whether renin-independent aldosteronism (i.e., subclinical PA), which fails to diagnose PA using current diagnostic criteria, is involved in cardiovascular disease. Elevated ARR, independent of brachial blood pressure, was associated with greater arterial stiffness and adverse cardiac remodeling, which was also observed in normotensive participants (140). Therefore, a low renin phenotype seems to be necessary to predict cardiovascular complications due to dysregulated aldosterone secretion.

In contrast, whether reversal of renin suppression ameliorates the excess risk of cardiovascular complications due to dysregulated aldosterone secretion should be investigated. Several studies have demonstrated that in patients with PA, adrenalectomy and MRA can ameliorate the unfavorable effects of excess aldosterone to achieve similar mortality rates in patients with essential hypertension (141, 142). Additionally, a recent large retrospective cohort study demonstrated that patients with APA that undergo surgical adrenalectomy had a significantly lower risk for cardiovascular events than patients with essential hypertension by 40% (6). In medically treated PA patients, the same investigators demonstrated different outcomes between the two subpopulations with unsuppressed or suppressed PRA. Surprisingly, the former showed an identical risk profile to that of essential hypertensives, whereas the latter showed an almost three times higher risk. A similar association was observed in the occurrence of atrial fibrillation (143). Therefore, we may need to start a renin check to estimate future cardiovascular risk due to dysregulated aldosterone secretion, which is also useful for monitoring surgical or medical treatment efficacy in patients with PA.

## Optimal screening strategy for PA

As observed in other endocrine disorders (e.g., hyperthyroidism and hyperparathyroidism), the dysregulated hormones are not always beyond the normal range, and the hormone-receiving feedback loop is more sensitive in reflecting the disease. This may also be true for patients with PA. PA disrupts the homeostatic feedback loop between aldosterone and salt status (136). As we have overviewed, PA is a disease with a spectrum, and inappropriate aldosterone secretion increases gradually. Furthermore, aldosterone secretion is affected by diurnal variations and salt sensitivity, which vary widely between individuals. Additionally, aldosterone driver mutations, such as *KCNJ5* and *CACNA1D* mutations, significantly affect aldosterone secretion, with large sex and ethnic differences. Therefore, rather than setting a certain threshold for aldosterone levels to detect PA, using renin suppression as a feedback loop for inappropriate aldosterone secretion early in its natural history is reasonable. However, establishing a clear threshold for PRA suppression is challenging. Therefore, we should begin with the values used in the current guidelines (PRA <1.0 ng/mL/h) as a standard to accumulate further knowledge.

**Figure 1:**
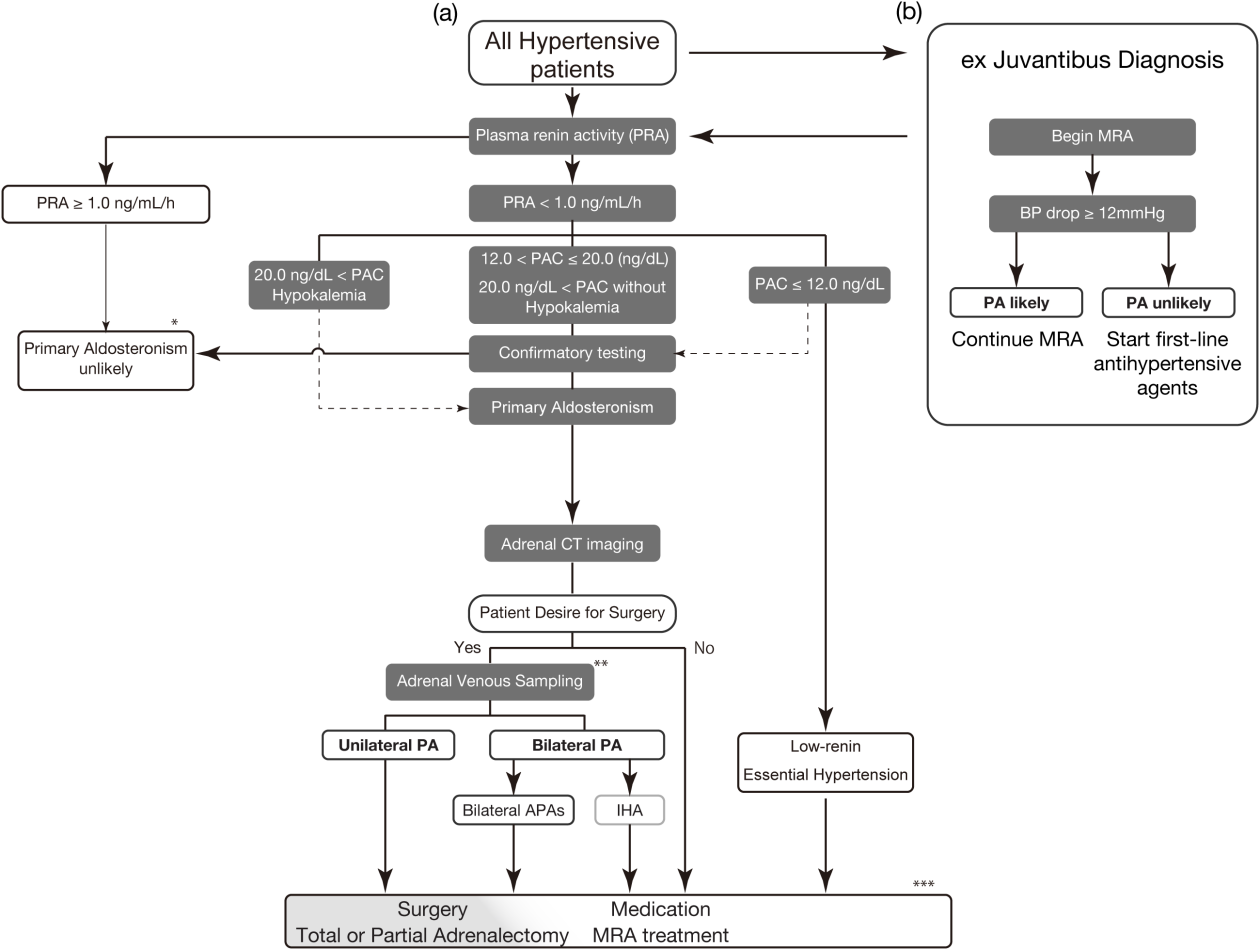
Proposed treatment strategy for primary aldosteronism **(A)** The screening for PA in all hypertensive patients should begin with PRA evaluation. Withdrawal of interfering antihypertensive drugs is preferable. However, many medications and conditions do not significantly hinder the detection of typical PA. RIA is used for PAC values in this figure. Low-renin indicates PRA <1.0 ng/mL/h. Physicians should perform confirmatory testing for cases with PAC of less than 12.0 ng/dL if PA is clinically suspected. Confirmatory testing options include the oral sodium loading test, captopril challenge test, saline infusion test, and 24-h urinary aldosterone excretion (>12 µg/24 h) after sodium intake correction (9). **(B)** If the proper evaluation of renin is difficult due to interfering factors, proceed with ex juvantibus diagnosis. Begin administration of MRA (e.g., spironolactone 25 mg/day) for 4 weeks to see whether there is a drop in blood pressure. A drop of 12 mmHg or more is suspected of a high likelihood of PA. * Reconsider the diagnosis if the case has the factors increasing renin (e.g., excess cortisol secretion, chronic kidney disease, nephrotic syndrome, liver dysfunction, and chronic heart failure) ** Consider segment-selective AVS if the clinical diagnosis and conventional AVS diagnosis are inconsistent. *** Partial adrenalectomy is a treatment option for cases with bilateral APAs. PAC, plasma aldosterone concentration; PRA, plasma renin activity; CT, computed tomography; PA, primary aldosteronism; APA, aldosterone-producing adenoma; IHA, idiopathic hyperaldosteronism; MRA, mineral corticoid receptor antagonist.

We propose that individual hormone levels of renin and aldosterone can help diagnose PA (**Figure 1A**). We demonstrated the prevalence of PA at a general outpatient clinic in 2004, where endocrine markers of secondary hypertension, such as renovascular hypertension, Cushing’s syndrome, and pheochromocytoma, were evaluated (22). We used PAC_RIA_ (>12 ng/dL) and PRA (<1.0 ng/mL/h) individually for screening in this study, finding a prevalence of PA of 6.0%, which is consistent with a recent report in a primary care setting (30). For individuals exhibiting a low-renin phenotype, a PAC_CLEIA_ exceeding 10 ng/dL (or equivalently, a PAC_RIA_ greater than 20 ng/dL) or the presence of hypokalemia (serum potassium less than 3.5 mEq/L) may be sufficient to diagnose PA without the need for further confirmatory testing (9). Notably, a few patients with PA show high plasma renin levels due to comorbidity (144) (such as excess cortisol secretion, chronic kidney disease, nephrotic syndrome, liver dysfunction, and chronic heart failure).

In settings in which proper evaluation of reninemia is not feasible, MRA is a useful strategy for ex Juvantibus diagnosis (**Figure 1B**). Renin is more sensitive than the ARR for detecting PA (8, 20, 145). A low-renin phenotype indicates extracellular fluid volume expansion or an MR-activated state (146–149). In patients with hypertension but without a PA diagnosis, those with suppressed renin levels experience a greater blood pressure reduction from MRA treatment, particularly if they have higher plasma aldosterone levels within the normal range (150, 151). This suggests that such patients may represent a wider spectrum of potential patients with PA (6, 141, 142, 152–154). We referred to a recent Commentary from Dr. Funder (155), who proposed to begin the administration of spironolactone 25 mg/day for 4 weeks and measure the blood pressure response. In hypertensives, a drop of less than 10 mmHg indicated a low probability of PA, whereas a drop of 12 mmHg or more suggested a high likelihood of PA. The same applies to newly developed hypertension, where spironolactone is prescribed 25 mg/day for 4 weeks. If blood pressure falls within the normal range, continue; otherwise, prescribe first-line antihypertensive agents. These steps are an effective strategy to ensure that as many patients with PA as possible receive the necessary medical treatment, regardless of the medical environment. Treatment with MRA carries certain risks, such as hyperkalemia and a decline in glomerular filtration rate. Occasionally, MRA may not effectively reverse renin suppression. In such situations, or if the patients wish to explore the possibility of curative treatment, they should be referred to an appropriate specialized center for reevaluation of the diagnosis of PA.

The perceptions of primary care physicians who see patients with PA are also critical for lowering the hurdles for PA screening. Actions are needed to increase knowledge of PA among these physicians, including its high prevalence and minor presentation of hypokalemia. The rapid immunoassay for plasma aldosterone and renin may lessen the hurdle for their measurement and encourage screening procedures (69). This will contribute to an increase in the population diagnosed with PA by more than >1% (156).

## Perspectives

To design a better screening method, we addressed the following questions: 1) Is early intervention for normotensive renin-independent aldosteronism beneficial for the patient’s prognosis? 2) What is the cutoff for PRA and DRC to stratify the population according to excess cardiovascular risk due to hyper-aldosteronism? 3) What are the most cost-effective screening methods? 4) What is the clinically helpful definition of renin-independent aldosteronism and essential hypertension and vice versa? These answers will help us design a better screening algorithm for PA. Additionally, we need evidence that the algorithm can identify all cases that benefit from PA treatment at an early stage. Finally, we emphasize that evidence using the PAC value by CLEIA is warranted. Accumulated clinical data from larger samples will facilitate the development of a new screening strategy for PA.
